# Special Issue “Membrane Catalysis”

**DOI:** 10.3390/molecules21070851

**Published:** 2016-06-28

**Authors:** Raffaele Molinari

**Affiliations:** Department of Environmental and Chemical Engineering, Università della Calabria, 87036 Rende (CS), Italy; raffaele.molinari@unical.it; Tel.: +39-0984-496699

**Keywords:** palladium-silver alloy, H_2_ separation, fluidized bed membrane reactor, water gas shift, Pd-Au membrane, methane steam reforming, composite membrane, hydrogen production, immobilized lipase, ceramic biocatalytic membrane, two-separate-phase biocatalytic membrane reactor, catalytic membranes, ketone hydrogenation, zeolite membranes, catalytic wet oxidation, formaldehyde, mixed Ce-Co membrane

Membrane technology is recognized as a scientific sector of multidisciplinary interest. In this context, the coupling of Membrane and Catalysis motivated this Special Issue.

In many cases, the potentialities of membrane processes and those of catalytic processes are enhanced thanks to their synergy. Membrane Catalysis is performed in a device called Membrane Reactor (MR), where the chemical reaction and the separation process can be accomplished simultaneously in the same physical device, thus fulfilling the criteria of process intensification and minimizing environmental and economical impacts. Generally the membrane allows the confinement of the catalyst in the reaction ambient, thus facilitating its reuse and also permitting the selective separation of specific molecules present in the reaction ambient. As a result, a minimization of the formation of by-products, thus improving conversion, selectivity, and yield, can be obtained. Higher energy efficiency, modularity, and easy scale-up are some other advantages of Membrane Catalysis compared to conventional catalysis. The appropriate choice of the membrane type, membrane module configuration, and MR is mainly determined by the type of catalysis (e.g., homogeneous, heterogeneous, photo, bio) where the membrane can assume many roles in catalyst recovery, separation of the products, rejection of the substrate, etc.

In this Special Issue, six original research articles covering some of the most recent advances in Membrane Catalysis of basic interest or relevant for applications are reported. Three articles deal with the production and purification of hydrogen which is a very relevant application of membranes and membrane reactors. Fernandez et al. [1] report on the preparation of Pd-Ag films deposited on ZrO_2_ nanoparticles to be used for the purification of H_2_. They studied the influence of the temperature during the growth of Pd-Ag films by Physical Vapor Deposition (PVD) magnetron sputtering onto polished silicon wafers in order to avoid the effect of the support roughness on the layer growth. Helmi et al. [2] studied the performance of a fluidized bed membrane reactor for a high temperature water-gas shift and its long-term stability to provide a proof-of-concept of the new system at lab scale. They obtained high hydrogen recovery factors and very stable performance of the membranes and the reactor in continuous operation. Iulianelli et al. [3] studied a supported Pd-Au membrane produced by electroless plating deposition which was allocated in a membrane reactor module for a methane steam reforming reaction, finding 35% hydrogen recovery using a commercial Ni/Al_2_O_3_ catalyst. The use of Membrane Catalysis in the biocatalytic field has been studied by Ranieri et al. [4]. They prepared asymmetric, ceramic, hollow fiber membranes which were then used as a support for the covalent immobilization of lipase in order to develop a two-separate-phase biocatalytic membrane reactor. Results showed that it is possible to immobilize lipase on a ceramic membrane without altering its catalytic performance. Membrane and photocatalysis has been studied by Molinari et al. [5] which prepared and tested Pd-loaded hierarchical Faujasite Pd-FAU) membranes, containing an intrinsic secondary non-zeolitic (meso)porosity in the catalytic transfer hydrogenation of acetophenone (AP) to produce phenylethanol (PE), an industrially relevant product. The Pd-loaded FAU membrane showed enhanced catalytic performance compared to the unsupported Pd-FAU crystals. Gutiérrez-Arzaluz et al. [6] report the synthesis of cerium oxide, mixed cerium and cobalt oxides and a Ce-Co/Al_2_O_3_ membrane, which were employed as catalysts for the catalytic wet oxidation of formaldehyde from industrial effluents.

In conclusion, the research area of Membrane Catalysis is growing and some applications seem very promising. I thank all of the authors for their contributions to this Special Issue and the staff members of MDPI for the editorial support.
